# ETC-1002 Attenuates *Porphyromonas gingivalis* Lipopolysaccharide-Induced Inflammation in RAW264.7 Cells *via* the AMPK/NF-*κ*B Pathway and Exerts Ameliorative Effects in Experimental Periodontitis in Mice

**DOI:** 10.1155/2022/8583674

**Published:** 2022-03-16

**Authors:** Hongyan Li, Peipei Zhang, Hongbing Lin, Huan Gao, Jianyuan Yin

**Affiliations:** ^1^School of Pharmaceutical Sciences, Jilin University, Changchun 130021, China; ^2^Hospital of Stomatology, Jilin University & Jilin Provincial Key Laboratory of Oral Biomedical Engineering, Changchun 130021, China; ^3^Department of Pharmacy, The First Hospital of Jilin University, Changchun 130021, China

## Abstract

**Background:**

Clinically, the failure of periodontal therapy stems largely from an inability to control the inflammatory response. Resolution of inflammation is an active, energy-requiring repair process, not merely a passive termination of inflammation. AMP-activated protein kinase (AMPK), a key energy sensor, has been shown to negatively regulate inflammatory signaling pathways. Thus, there is a crucial need for new therapeutic strategies to modulate AMPK and to promote enhanced resolution of inflammation. This study is aimed at investigating the anti-inflammatory effects of ETC-1002 through modulating AMPK in periodontitis.

**Methods:**

RAW264.7 cells were infected with *Pg*-LPS in the presence or absence of ETC-1002, following which the expression levels of proinflammatory cytokines and inflammation signaling-related proteins were evaluated by real-time reverse transcription-quantitative polymerase chain reaction (RT-qPCR) and western blotting. ETC-1002 was applied in a murine model of periodontitis to determine its anti-inflammatory effect *in vivo*. Histological changes were investigated by hematoxylin and eosin (H&E) staining, the levels of proinflammatory cytokines were detected using immunohistochemistry, and alveolar bone height was measured using micro-CT imaging.

**Results:**

ETC-1002 inhibited the production of proinflammatory cytokines, promoted AMPK phosphorylation, and decreased I*κ*B*α* and NF-*κ*B p65 phosphorylation levels in *Pg*-LPS-treated RAW264.7 macrophages. The inhibitory effects of ETC-1002 on the production of proinflammatory mediators were significantly abrogated by siRNA-mediated silencing of AMPK*α* in RAW264.7 cells. *In vivo*, ETC-1002 inhibited inflammatory cell infiltration, the expression of proinflammatory cytokines, and the inflammation-mediated destruction of alveolar bone in mice with experimental periodontitis. The anti-inflammatory effect of ETC-1002 in the periodontium could be reversed by the administration of Compound C, an AMPK inhibitor.

**Conclusions:**

ETC-1002 exerts anti-inflammatory effects in *Pg*-LPS-treated RAW264.7 cells *via* the AMPK/NF-*κ*B pathway *in vitro* and inhibits the progress of experimental periodontitis in mice in an AMPK signaling-dependent manner *in vivo*. These results provide evidence for the beneficial effects of ETC-1002 in the treatment of periodontitis.

## 1. Introduction

Periodontitis is a chronic inflammatory disease of the periodontium [1, 2] that leads to irreversible alveolar bone destruction and tooth loss [3]. Periodontitis not only affects masticatory function and aesthetics but is also interrelated with systemic health through increasing the risk of several disorders, including cardiovascular disease, diabetes mellitus, rheumatoid arthritis, cancer, and Alzheimer's disease [4–6]. The treatment of periodontitis is aimed at reducing and eliminating plaque microorganisms through mechanical methods or the use of antibiotics. Although these treatments can alleviate periodontal inflammation and slow the progress of the disease [7, 8], the success of these operations is not highly predictable in all patients, and complete and reliable periodontal regeneration is not feasible at present. Failure of periodontal therapy stems largely from an inability to control the associated inflammation [9]. Chronic inflammation is accompanied by changes in energy metabolism [10]. Resolution of inflammation is an active, energy-dependent process and does not merely involve the passive termination of inflammation. It will be more promising to solve inflammation from the perspective of energy repair.

Macrophages play an important role in innate immunity for the first line of host defense against microorganisms and are one of the major sources of the destructive cytokines, such as interleukins (IL-1*β*, IL-6) and tumor necrosis factor (TNF-*α*), which are critical for the inflammatory response that exacerbates tissue damage in periodontitis [11]. Macrophage-mediated inflammation is closely related to a reduction in AMP-activated protein kinase (AMPK) activity [12–15]. AMPK is the most important energy sensor in cells [16]. When activated, AMPK inhibits energy-consuming anabolic processes and enhances catabolism, leading to an increase in adenosine triphosphate (ATP) levels in cells, thereby helping to maintain cellular energy and allowing for the repair of inflammation-related injury [17]. The activation of AMPK by phosphorylation has been shown to negatively regulate the nuclear factor kappa B- (NF-*κ*B-) dependent inflammatory signaling pathway both *in vivo* and *in vitro* [18–20]. AMPK activation prevented alveolar bone resorption and periodontal inflammation induced by ligature placement in rat periodontitis model and decreased the expression of *Pg*-LPS-induced inflammatory cytokines in human gingival fibroblast cells [21–23]. Consequently, there is a crucial need for new therapeutic strategies to modulate AMPK and to promote enhanced resolution of inflammation in periodontitis.

ETC-1002 (8-hydroxy-2,2,14,14-tetramethylpentadecanedioic acid), previously known as ESP55016 or bempedoic acid, is a novel drug under development for the treatment of dyslipidemia. ETC-1002 has been shown to improve lipid profiles and significantly attenuate levels of glucose *via* the activation of AMPK in multiple animal models [24, 25]. Moreover, ETC-1002 was found to regulate immune responses and adipose tissue inflammation *via* the LKB1-dependent activation of AMPK in macrophages [26]. These observations suggest that ETC-1002 may have a significant effect on the pathology of periodontitis *via* the AMPK pathway.

**Scheme 1:**
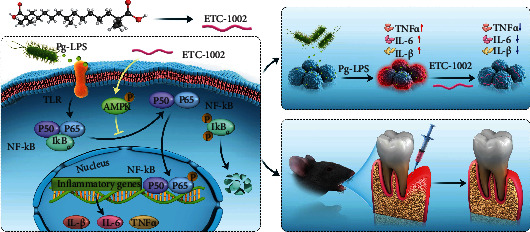
Schematic illustration of the anti-inflammatory effect and the working mechanism of ETC-1002 in periodontitis.

Here, we investigated the anti-inflammatory effects of ETC-1002 in *Porphyromonas gingivalis* lipopolysaccharide- (*Pg-*LPS-) treated RAW264.7 macrophages. Our results showed that ETC-1002 exerted anti-inflammatory effects *via* the AMPK/NF-*κ*B signaling pathway. Moreover, in a murine model of periodontitis, we found that ETC-1002 treatment could efficiently inhibit inflammatory responses in the periodontium and promote the regeneration of collagen fibers and alveolar bone. Our findings indicated that the anti-inflammatory properties of ETC-1002 may offer clinical benefits for the treatment of patients with periodontitis (Scheme 1).

## 2. Materials and Methods

### 2.1. Cell Culture

The RAW264.7 murine macrophage cell line was purchased from the Shanghai Cell Bank of the Chinese Academy of Sciences. RAW264.7 cells were cultured in Dulbecco's modified Eagle's medium (DMEM; Invitrogen, Carlsbad, CA, USA) supplemented with 100 U/mL penicillin, 100 *μ*g/mL streptomycin (Invitrogen), and 10% fetal bovine serum (FBS; Invitrogen). The cells were incubated and maintained at 37°C with 5% CO_2_ and 100% humidity. The cells were passaged every 2 days, and cells in the exponential phase were used throughout the study.

### 2.2. Cell Viability Assay

ETC-1002 cytotoxicity against RAW264.7 cells was evaluated by Cell Counting Kit-8 (CCK-8) assay (Beyotime Biotechnology, Shanghai, China). Briefly, cells (5 × 10^3^ cells/well) were seeded in a 96-well culture plate and treated with different concentrations of ETC-1002 (25, 50, 100, 200, and 400 *μ*M) for 24 and 48 h. The culture medium was subsequently replaced with serum-free DMEM containing 10% CCK-8 reagent and incubated at 37°C for 4 h. The optical density at 450 nm was determined using a microplate reader. The percent cell activity was determined as the absorbance value of the experimental group/absorbance value of the control group × 100%. The experiments were performed in triplicate. The concentrations of ETC-1002 used in subsequent experiments were selected based on the results of the assay.

### 2.3. Cell Model of Inflammation-Induced Injury and Grouping

RAW264.7 cells were seeded in a 6-well plate (5 × 10^5^ cells/well) and incubated for 24 h. After incubation with or without ETC-1002 (50 or 100 *μ*M) (MCE, NJ, USA, Catalog number: 738606-46-7) for 1 h, the cells were treated with *Pg*-LPS (Sigma, St Louis, MO, USA) (10 *μ*g/mL) for 12 h and then harvested and stored at −80°C for RT-qPCR and western blot assays. Untreated cells served as controls. To further identify the role of the AMPK pathway in the anti-inflammatory effects of ETC-1002, siRNA targeting AMPK*α* was also added.

### 2.4. ELISA

The levels of IL-6, IL-1*β*, and TNF-*α* secreted by the RAW264.7 cells were detected using ELISA kits (Boster Biological Technology, California, USA): mouse IL-1*β* ELISA kit (EK0394), mouse TNF-*α* ELISA kit (EK0527), and mouse IL-6 ELISA kit (EK0411).

### 2.5. siRNA-Mediated Silencing of AMPK*α* in RAW264.7 Cells

siRNA oligonucleotides (20 nM) specifically targeting AMPK*α* were obtained from Santa Cruz Biotechnology (Santa Cruz, CA, USA). RAW264.7 cells were grown to 60%–80% confluence, serum-starved for 12 h, and then transfected for 5-7 h with target-specific siRNA or scramble siRNA at the final concentrations of 20 nM in the presence of Lipofectamine 2000 (Invitrogen) according to the manufacturer's instructions. After transfection, the cells were allowed to recover for 24 h in normal growth medium before treatment with *Pg*-LPS and/or ETC-1002.

### 2.6. RNA Extraction and Real-Time Reverse Transcription-Quantitative Polymerase Chain Reaction (RT-qPCR) Assays for the Detection of Proinflammatory Cytokine Levels

*In vitro* anti-inflammatory effects of ETC-1002 were assessed by examining expression of proinflammatory cytokine (IL-1*β*, IL-6, and TNF-*α*) using RT-qPCR assays. Total RNA was extracted from harvested RAW264.7 cells using TRIzol reagent (Life Technologies, Carlsbad, CA, USA) and reverse transcribed into cDNA using Hifair II 1st Strand cDNA Synthesis Super Mix (Yeasen Biotech, Shanghai, China). qPCR was performed with Hieff qPCR SYBR Green Master Mix (Yeasen Biotech) following the manufacturer's instructions. The primer sequences used for RT-qPCR are described in Table 1.

### 2.7. Western Blot Analysis

Total protein was extracted from RAW264.7 cells with RIPA lysis buffer (Saint-Bio, Shanghai, China) containing 1% phenylmethanesulfonyl fluoride (PMSF). After centrifugation at 12,000 × *g* for 5 min at 4 °C, protein concentrations in the collected supernatants were quantified using the BCA method. Equal amounts of protein were mixed with 5x loading buffer, heated for 5 min at 95°C, separated by 8% and 12% sodium dodecyl sulfate–polyacrylamide gel electrophoresis (SDS-PAGE), and transferred onto polyvinylidene difluoride (PVDF) membranes (Millipore, Massachusetts, USA). After blocking with 5% skimmed milk, the membranes were first incubated with primary antibodies targeting phosphorylated p-AMPK*α*1/2 (1 : 1000; #2531), AMPK*α*1/2 (1 : 2500; #2532), p-NF-*κ*B p65 (1 : 1000; #3033), p-I*κ*B*α* (1 : 1000; #2859), I*κ*B*α* (1 : 1000; #4814) (all from Cell Signaling Technology, Boston, USA), NF-*κ*B p65 (1 : 2500; #10745-1-AP), IL-6 (1 : 1000; #66146-1-Ig), IL-1*β* (1 : 1000; #26048-1-AP), and TNF-*α* (1 : 1000; #17590-1-AP) (all from Proteintech, Chicago, IL, USA) at 4°C overnight and then with the corresponding horseradish peroxidase-conjugated secondary antibodies for 1 h. Relative protein band intensities were detected using an enhanced chemiluminescence (ECL) reagent and quantified using ImageJ software.

### 2.8. Animals

Specific-pathogen-free (SPF) female C57BL/6 mice aged 10-11 weeks and weighing 20-22 g [27] were obtained from the Experimental Animal Center of the Shanghai Branch of the Chinese Academy of Sciences. The mice were housed under standard controlled conditions (temperature 25 ± 1°C, 12 : 12 h light/dark cycle) and had free access to food and water. The animals were allowed to adapt to the environment for one week before the experiment. All operations were performed under anesthesia, and all efforts were made to minimize the suffering of the animals. All the animal experiments were carried out in accordance with the guidelines of the Institutional Animal Care and Use Committee of Jilin University (approval number: SY202105004).

### 2.9. Mouse Model of Experimental Periodontitis

A sterile silk suture ligature was prepared by cutting a 7- to 10-inch long silk thread and tying two knots in the center, 2.5 mm apart. The ligature was placed between the first and second molars (M1/M2) in the right maxillary bone of anesthetized mice (injection with sterile ketamine/xylazine) and cut as close as possible to the knots [27]. The mice were subsequently placed in a sterile cage with a heat lamp until they had fully recovered from the anesthesia and then moved from the surgical room to the housing room and housed under SPF conditions for 10 days to induce a periodontal phenotype.

### 2.10. Experimental Protocols

A total 24 of mice were randomly divided into the following four groups (*n* = 6 per group): a control group, comprising healthy mice with no ligation but treated with phosphate-buffered saline (PBS); a periodontitis group, comprising mice with ligature-induced periodontitis and treated with PBS; an ETC-1002 group, consisting of mice with ligature-induced periodontitis and treated with ETC-1002 (100 *μ*M, 5 *μ*L); and an ETC-1002+Compound C group, consisting of mice with ligature-induced periodontitis and treated with ETC-1002 (100 *μ*M, 5 *μ*L) and Compound C (10 mg/kg).

Concomitant with the establishment of the mouse model of experimental periodontitis, 5 *μ*L of ETC-1002 (100 *μ*M) (MCE) was injected into the distal alveolar crest of the first molar by microsampler every other day. The mucosa was white and without obvious exudation during the injection. After injection, the microsampler was indwelled locally for 1 min.

Compound C (10 mg/kg) (MCE) was intraperitoneally injected 30 min before ETC-1002 injection [28]. Ten days later, three mice per group were anesthetized and euthanized by perfusion fixation with 4% paraformaldehyde (PFA; Coolaber Science & Technology, Beijing, China). Part of the maxillae, including the maxillary first and second molars, together with the alveolar bone was collected and fixed in 4% PFA for 2 days for micro-computed tomography (micro-CT) imaging, hematoxylin and eosin (H&E) staining, and immunohistochemical (IHC) staining. The other three mice in each group were euthanized, and the periodontium of maxillary first molars was immediately placed in liquid nitrogen for RT-qPCR. All the ligatures were removed.

### 2.11. Micro-CT and Analysis

The samples were washed with PBS (Gibco, New York, USA) and subjected to micro-CT (*μ*CT35, Scanco Medical AG, Bassersdorf, Switzerland) in a 19 mm diameter tube (12 *μ*m voxel size, 114 mA, 70 kVp, and 300 ms exposure time). For the assessment of alveolar bone loss, the distance between the cementoenamel junction and the alveolar bone crest (CEJ-ABC) for the distal buccal root of the first molars was measured in 3D images viewed from the buccal side, as previously described [27]. The measurements were repeated three times per site, and mean distances in millimeters were obtained.

### 2.12. H&E Staining

Fixed maxillaries were decalcified in a 10% ethylenediaminetetraacetic acid (EDTA; Beijing Chemical Works, Beijing, China) (pH 8.0) solution at 4°C for 2 weeks (the EDTA solution was changed every other day). The samples were rinsed for 24 h and dehydrated in a series of graded ethanol solutions, embedded in paraffin (the buccal side of the tooth facing the bottom of the micromold and the long axis of the tooth parallel to the short side of the box), and cut into 5 *μ*m thick sections [27]. The slices were dewaxed with xylene, rehydrated in a graded alcohol series (100%, 90%, 80%, and 70%), and subjected to H&E staining. The inflammatory response and histological changes in the periodontium were evaluated under an optical microscope.

### 2.13. IHC Staining

Tissue sections were baked in an oven at 65°C, deparaffinized in xylene, hydrated *via* a graded ethanol series, dipped in boiling citric acid buffer (pH 6.0), and treated with 3% hydrogen peroxide to inactivate endogenous peroxidase activity. After blocking in 1% bovine serum albumin (BSA) for 1 h, the slides were first incubated with primary antibodies (from Proteintech) against TNF-*α* (1 : 100, #0291-1-Ig), IL-1*β* (1 : 100, #16806-1-AP), and IL-6 (1 : 100, #66146-1-Ig) overnight at 4°C and then with secondary antibody. The slides were washed three times with PBS, diaminobenzidine hydrochloride (DAB; Dako, Carpinteria, CA, USA) was added as the substrate, and the nuclei were counterstained with hematoxylin. Finally, the tissue was differentiated in hydrochloric acid alcohol solution, placed in saturated lithium carbonate until the specimens turned blue, dehydrated in a graded ethanol series, made transparent with xylene, and sealed with neutral gum. For each group, the average optical density (AOD) was determined in each of three randomly selected discrete fields of vision (×400 magnification) using ImageJ software. When the amount of target substance to be measured is greater, the optical density (OD) is higher. Add up the optical density values of each positive point on the picture to obtain IOD. The IOD value was divided by the area of the distribution area of the target substance to obtain average optical density (AOD), which reflects the unit area concentration of the target substance. The “staining scores” in the figure are the mean of AOD of all the three groups.

### 2.14. RNA Extraction from Mouse Periodontal Tissue and RT-qPCR Assay for Proinflammatory Cytokine Levels

Total RNA was extracted from mouse periodontal tissue and reverse transcribed into cDNA for measuring the relative expression levels of IL-6, TNF-*α*, and IL-1*β* by qPCR. The experimental procedures and primer sequences for IL-6, TNF-*α*, IL-1*β*, and *β*-actin amplification were the same as those described in RNA Extraction and Real-Time Reverse Transcription-Quantitative Polymerase Chain Reaction (RT-qPCR) Assays for the Detection of Proinflammatory Cytokine Levels.

### 2.15. Statistical Analysis

All data were analyzed using GraphPad Prism 8.0 software and presented as means ± standard deviation (SD). All experiments were performed at least three times. The Student's *t*-test was used for comparisons between two groups, and analysis of variance (ANOVA) was used for comparisons among multiple groups. Significance was accepted at *P* < 0.05.

## 3. Results

### 3.1. ETC-1002 Inhibited the Release of Proinflammatory Cytokines in Pg-LPS-Treated RAW264.7 Cells

**Figure 1:**
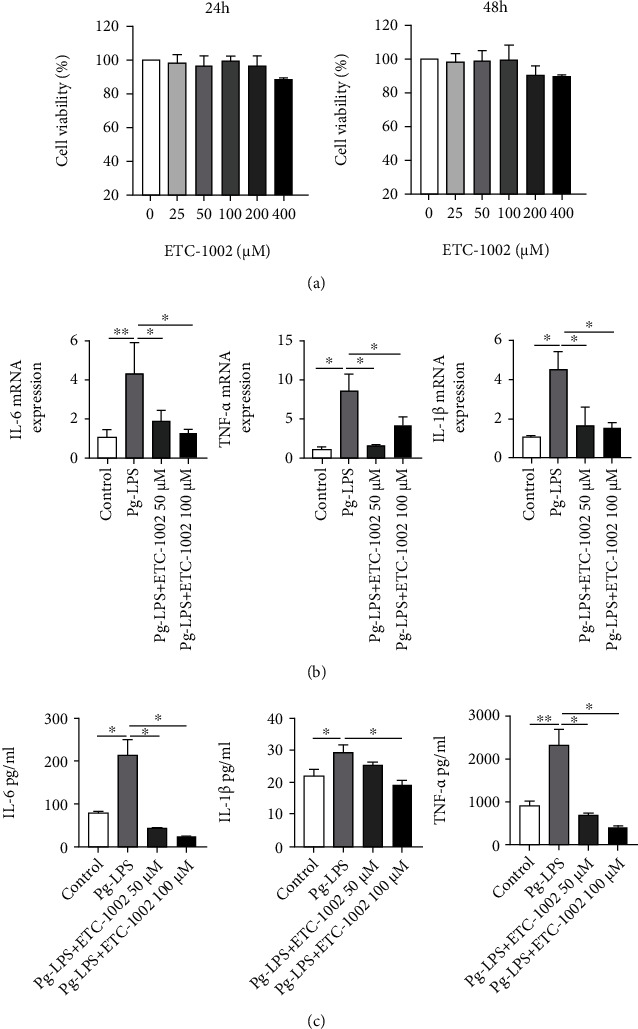
ETC-1002 suppresses *Pg*-LPS-induced inflammation in RAW264.7 cells. (a) Cytotoxic effect of ETC-1002 at different concentrations on RAW264.7 for 24 h or 48 h. (b) qRT-PCR analysis of IL-6, IL-1*β*, and TNF-*α* mRNA expression in *Pg*-LPS (10 *μ*g/mL)-treated RAW264.7 cells with or without ETC-1002 (50, 100 *μ*M) for 12 h. (c) ELISA analysis of IL-6, IL-1*β*, and TNF-*α* protein level in *Pg*-LPS (10 *μ*g/mL)-treated RAW264.7 cells with or without ETC-1002 (50, 100 *μ*M) for 12 h. ^∗^*P* < 0.05 and ^∗∗^*P* < 0.01.

No significant differences in phenotype were observed among cells treated with different concentrations of ETC-1002 for 24 h or 48 h as determined by CCK-8 assay, suggesting that ETC-1002 has good biocompatibility *in vitro* (Figure 1(a)). Consequently, 50 and 100 *μ*M were selected as the experimental concentrations based on the existing literature [26].

To investigate the anti-inflammatory effects of ETC-1002 in periodontitis, RAW264.7 cells were treated with *Pg*-LPS (10 *μ*g/mL) in the presence or absence of varying concentrations of ETC-1002, following which the expression and secretion of proinflammatory cytokines were evaluated by RT-qPCR and ELISA, respectively. The results showed that the expression of the proinflammatory cytokines IL-1*β*, IL-6, and TNF-*α* remained at a low level in untreated cells. In contrast, stimulation with *Pg*-LPS triggered a marked increase in the expression of these cytokines; however, this increase was significantly attenuated following ETC-1002 treatment at both the mRNA (Figure 1(b)) and protein (ELISA) (Figure 1(c)) levels.

### 3.2. ETC-1002 Ameliorated Pg-LPS-Induced Inflammation in RAW264.7 Cells via the AMPK Signaling Pathway

**Figure 2:**
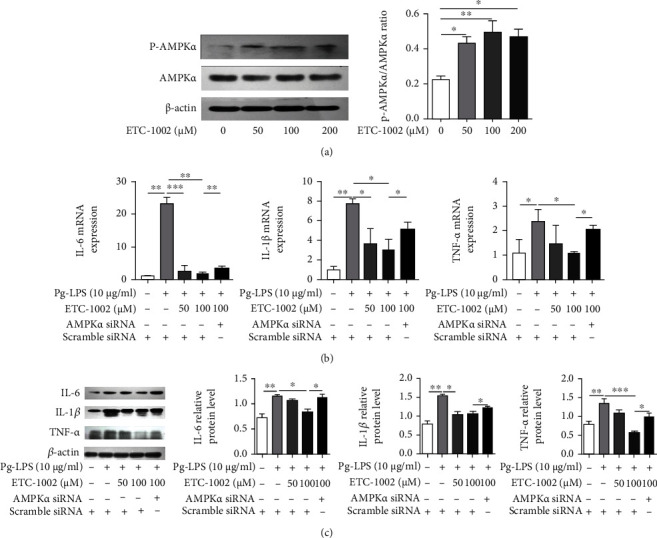
ETC-1002 ameliorates *Pg*-LPS-induced inflammation in RAW264.7 cells via AMPK-mediated pathway. (a) Western blot analysis of AMPK*α* phosphorylation in RAW264.7 cells treated with ETC-1002 (0-200 *μ*M) for 1 h. (b) mRNA expression of IL-6, IL-1*β*, and TNF-*α* in scramble or AMPK siRNA-transfected RAW264.7 cells treated with *Pg*-LPS (10 *μ*g/mL) and ETC-1002 (50, 100 *μ*M) for 12 h. (c) Western blot analysis of IL-6, IL-1*β*, and TNF-*α* in scramble or AMPK*α* siRNA-transfected RAW264.7 cells treated with *Pg*-LPS (10 *μ*g/mL) and ETC-1002 (50, 100 *μ*M) for 12 h. ^∗^*P* < 0.05, ^∗∗^*P* < 0.01, and ^∗∗∗^*P* < 0.001.

To determine whether the ETC-1002-mediated activation of AMPK yields anti-inflammatory effects, we first evaluated the effect of ETC-1002 on AMPK*α* phosphorylation in RAW264.7 cells. We found that ETC-1002 promoted AMPK*α* phosphorylation in a dose-dependent manner *in vitro* (Figure 2(a)). We next depleted the levels of AMPK*α* in *Pg*-LPS-treated RAW264.7 cells *via* siRNA-mediated knockdown and found that the suppressive effects of ETC-1002 on the expression of proinflammatory cytokines were significantly abrogated (Figures 2(b) and 2(c)). These results suggested that ETC-1002 treatment reduces the levels of proinflammatory cytokines in *Pg*-LPS-induced RAW264.7 cells *via* an AMPK-related pathway.

### 3.3. The AMPK/NF-*κ*B Pathway Contributes to ETC-1002-Mediated Anti-Inflammatory Effects in RAW264.7 Cells

**Figure 3:**
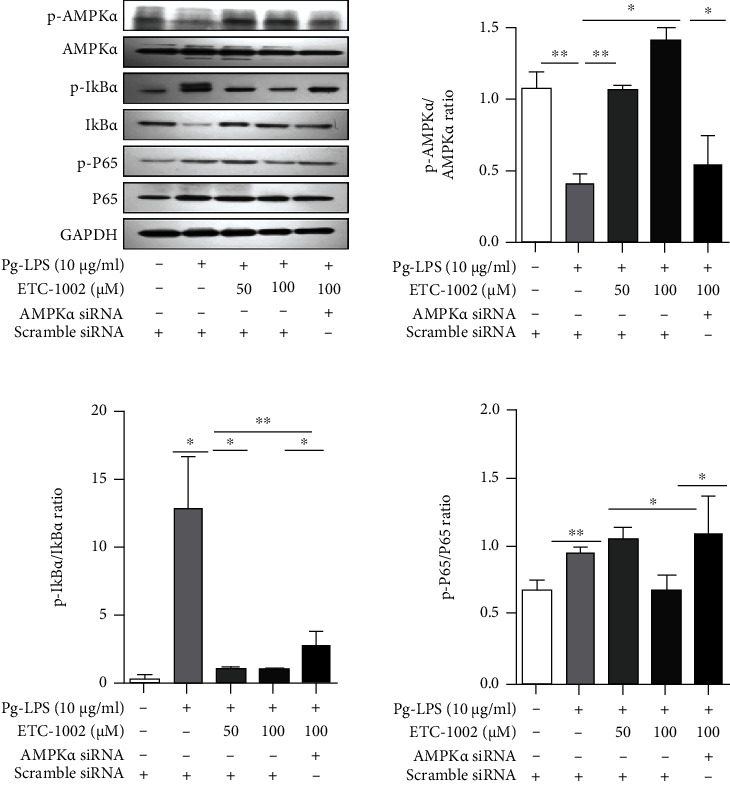
ETC-1002 promotes anti-inflammation via activation of AMPK pathway and inhibition of the NF-*κ*B pathway. Western blot analysis of AMPK*α*, p65, and I*κ*B*α* phosphorylation in scramble or AMPK*α* siRNA-transfected RAW264.7 cells treated with *Pg*-LPS (10 *μ*g/mL) and ETC-1002 (50, 100 *μ*M) for 12 h. ^∗^*P* < 0.05, ^∗∗^*P* < 0.01, and ^∗∗∗^*P* < 0.001.

When activated (phosphorylated), AMPK can inhibit NF-*κ*B signaling and inflammation. To test whether NF-*κ*B signaling is also involved in the anti-inflammatory effects of ETC-1002, we measured the expression levels of proteins associated with the AMPK/NF-*κ*B pathway in RAW264.7 cells in the different treatment groups by western blot assay. As seen in Figure 3, compared with the control condition, *Pg*-LPS treatment led to a decrease in the levels of p-AMPK*α*; notably, however, the addition of ETC-1002 resulted in a marked upregulation of AMPK*α* phosphorylation levels relative to the *Pg*-LPS group. Additionally, treatment with ETC-1002+AMPK*α* siRNA elicited opposing effects when compared with ETC-1002 treatment alone. These data indicated that ETC-1002 can activate the AMPK signaling pathway under conditions of *Pg*-LPS-induced inflammation.

Next, we examined the expression of NF-*κ*B p65 and I*κ*B*α*, the main components of the NF-*κ*B pathway. NF-*κ*B p65 and I*κ*B*α* phosphorylation can serve as a readout for NF-*κ*B pathway activation. We found that *Pg*-LPS administration resulted in an increase in the levels of p-p65 and p-I*κ*B*α* compared with the control group (Figure 3(b)), whereas ETC-1002 treatment led to a decrease in the levels of the phosphorylated forms of both proteins relative to the *Pg*-LPS group (Figure 3(b)). Interestingly, however, in the presence of AMPK*α*-specific siRNA, ETC-1002 failed to upregulate the levels of p-p65 and p-I*κ*B*α* (Figure 3(b)). Overall, these findings supported that ETC-1002 exerts its anti-inflammatory effects by inhibiting the NF-*κ*B pathway *via* the phosphorylation and activation of AMPK.

### 3.4. ETC-1002 Inhibited Ligature-Induced Alveolar Bone Resorption: Micro-CT Observation and Measurement

**Figure 4:**
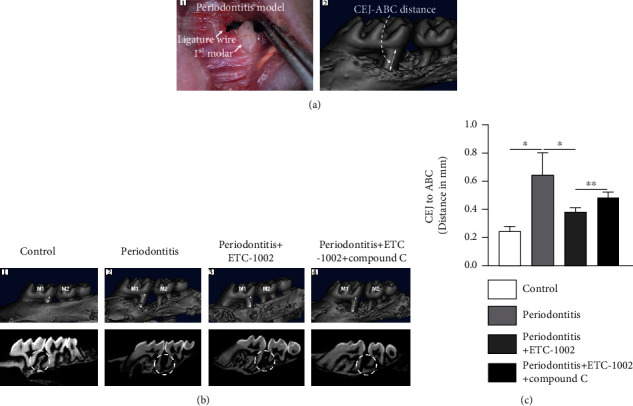
Three-dimensional micro-CT images of the alveolar bone between the maxillary first molar and second molar. (a1) A photograph of periodontitis model in C57BL/6 mice induced by ligature wire between the maxillary first molar and second molar. (a2) A scheme of the vertical distance between CEJ and ABC. (b) Micro-CT images of alveolar bone between the maxillary first molar (M1) and second molar (M2) 10 days after the treatment: the images with dark blue background are three-dimensional micro-CT reconstruction images; the images with black background are representative sagittal micro-CT slices. (c) Quantitative analysis of the distance between CEJ and ABC. ^∗^*P* < 0.05 and ^∗∗^*P* < 0.01.

As we found that ETC-1002 exerted potent anti-inflammatory effects in *Pg*-LPS-treated RAW264.7 cells, we next evaluated whether ETC-1002 could promote similar effects *in vivo*, an important consideration for the treatment of periodontitis. To this end, we generated a mouse model of experimental periodontitis (Figure 4(a1)). Because it is difficult to achieve an effective dose in the periodontal infection site in a short time with systemic administration, ETC-1002 was administered by local injection in this study. Ten days after the first administration, the mice were euthanized and maxillary samples were collected and scanned by micro-CT. The amount of bone loss was determined by measuring the CEJ-ABC distance in the distal buccal root of the first molar in 3D images viewed from the buccal side (Figure 4(a2)). We detected alveolar bone loss and osteoclast activation between the first and second maxillary molars where the ligature had been installed. Typically, the buccal sides showed accelerated bone loss in the model group (Figures 4(b2) and 4(c)). The CEJ-ABC distance in this group was 2.6-fold greater than that of the control group (Figures 4(b1), 4(b2), and 4(c), *P* < 0.05), indicating that the periodontitis model had been successfully established. The CEJ-ABC distance in the ETC-1002 group was 58.9% than that in the periodontitis group (Figures 4(b2), 4(b3) and 4(c), *P* < 0.05); however, when Compound C, an AMPK inhibitor, was coadministered with ETC-1002, the CEJ-ABC distance was 1.27-fold greater than that seen in the ETC-1002 group (Figures 4(b3), 4(b4), and 4(c), *P* < 0.01). These results indicated that the inhibitory effects of ETC-1002 on alveolar bone resorption were mediated through AMPK.

### 3.5. ETC-1002 Attenuated Inflammatory Responses and Periodontal Tissue Destruction

**Figure 5:**
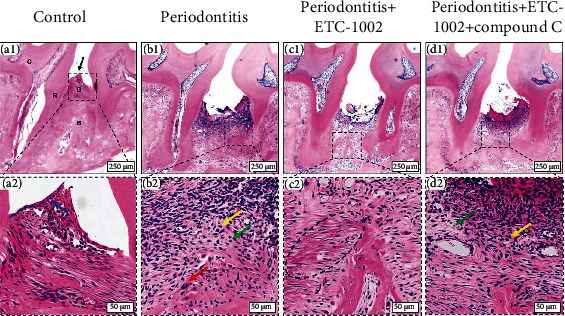
HE staining images of the periodontium 10 days after the treatment. Black arrow points to the interdental papillae, blue arrow points to the junctional epithelium, green arrows point to plasma cells, yellow arrows point to lymphocytes, red arrow points to the osteoclast, and black circle in (c2) points to the new reconstituted collagen fibrils.

To further confirm the above results, we evaluated the effects of ETC-1002 on the inflammation between the maxillary first and second molars using H&E staining. The control group had normal periodontal tissue structure (Figure 5(a)). The junctional epithelium (Figure 5(a2), blue arrow) adhered tightly to the surface of the enamel, and no erosion, ulceration, or inflammatory cell infiltration was found in the periodontium. On average, the ABC-CEJ distance was 0.22 ± 0.01 mm, indicating the absence of alveolar bone absorption. The fibers in the periodontal ligament were intact and arranged in an orderly manner (Figure 5(a2)). In contrast, the interdental papillae (Figure 5(a1), black arrow) had disappeared in the periodontitis group. Loss of connective tissue attachment and disordered periodontal fibers in the periodontium were evident between the distal root of the first molar and the mesial root of the second molar (Figure 5(b)). Meanwhile, the alveolar bone had resorbed, resulting in a significant increase in the ABC-CEJ distance (Figure 5(b)). These observations indicated that the experimental periodontitis model successfully mimicked the clinical symptoms of chronic periodontitis seen in humans. A large number of lymphocytes (Figure 5(b2), yellow arrow) and plasma cells (Figure 5(b2), green arrow) had infiltrated the periodontium. Osteoclasts could be clearly observed on the surface of alveolar bone (Figure 5(b2), red arrow). In the ETC-1002 group, the inflammatory response was continuously suppressed between the first and the second molars (Figure 5(c)), and relatively few and scattered inflammatory cells could be seen in the periodontium, likely due to the anti-inflammatory effects of ETC-1002. In the ETC-1002 group, we also found some collagen fibers, which were perpendicular to the tooth surface and regularly arranged, with one end being embedded in the alveolar bone and the other in the cementum (Figure 5(c2), black circle). Due to the anti-inflammatory effect of ETC-1002, these collagen fibers may be newly formed during repairment or undamaged by the inflammation. However, periodontal inflammation could again be detected with the coadministration of ETC-1002+Compound C (Figure 5(d)), providing further evidence that the anti-inflammatory effect of ETC-1002 was mediated through AMPK. The papilla and connective epithelium were not completely restored in any of the experimental groups due to the stimulation by the ligature throughout the experiment.

### 3.6. ETC-1002 Reduced the Expression of Proinflammatory Cytokines in Periodontal Tissue

**Figure 6:**
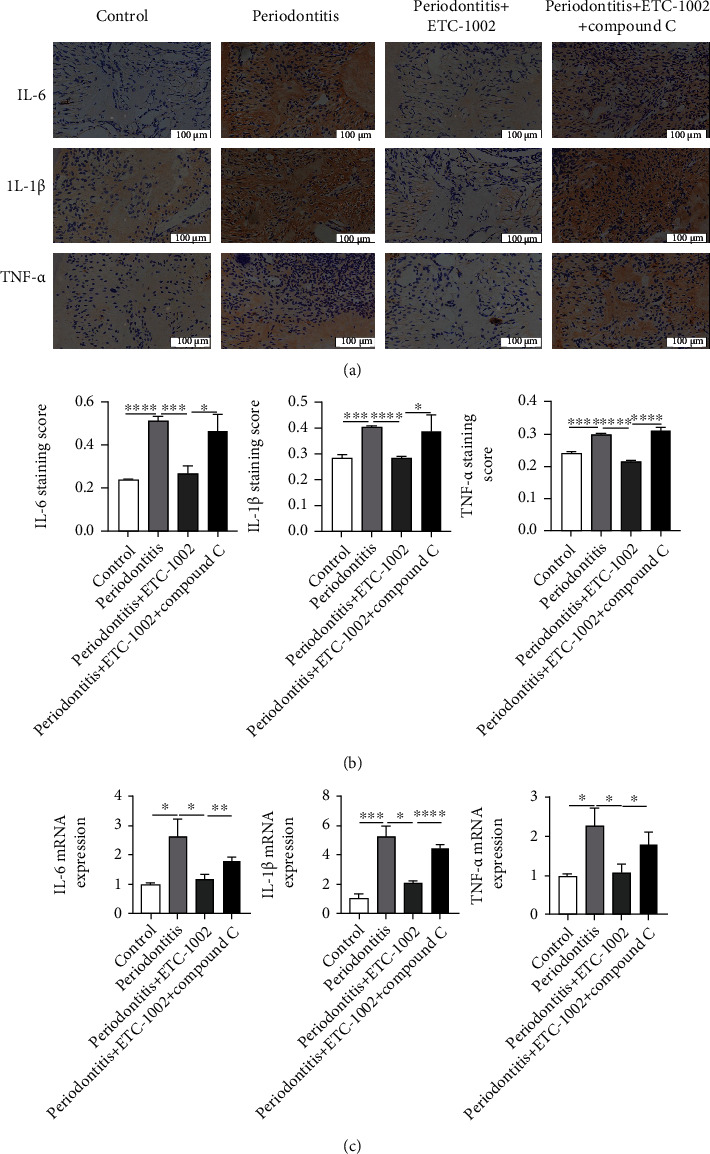
Anti-inflammation effects of ETC-1002 in periodontitis model in vivo. (a) IHC staining of IL-6, IL-1*β*, and TNF-*α* 10 days after treatment. (b) Relative density of IL-6-, IL-1*β*-, and TNF-*α*-positive area was measured in five fields (400x) located in 1 mm over the alveolar bone crest (between the maxillary first molar and second molar), and the averaged values were calculated. (c) Relative mRNA expression of IL-6, IL-1*β*, and TNF-*α* in the periodontium from the four groups. ^∗^*P* < 0.05, ^∗∗^*P* < 0.01, ^∗∗∗^*P* < 0.001, and ^∗∗∗∗^*P* < 0.0001.

To confirm that ETC-1002 can inhibit periodontal inflammation, the intensity of the inflammatory reaction was evaluated according to the density of IL-6-, IL-1*β*-, and TNF-*α*-positive areas in immunostained sections. IL-6, IL-1*β*, and TNF-*α* are normally expressed by cells in periodontal tissue, and this expression is concentrated in the cytoplasm and extracellular matrix (ECM). In the control group, only weak signals for IL-6, IL-1*β*, and TNF-*α* were detected in the periodontium, indicative of the absence of inflammation (Figure 6(a)). In contrast, IL-6, IL-1*β*, and TNF-*α* exhibited strong staining in the periodontitis group (Figure 6(a)), suggesting that the periodontitis model had been well established. IL-6, IL-1*β*, and TNF-*α* staining intensity was decreased in the ETC-1002 treatment group (0.27 ± 0.03, *P* < 0.001; 0.29 ± 0.003, *P* < 0.0001; and 0.22 ± 0.005, *P* < 0.0001, respectively) compared with that in the model group (0.51 ± 0.02, 0.41 ± 0.003, and 0.30 ± 0.003, respectively) (Figures 6(a) and 6(b)), indicating that ETC-1002 can effectively inhibit periodontal inflammation. Importantly, IL-6, IL-1*β*, and TNF-*α* staining intensity was greater in the ETC-1002+Compound C cotreatment group (0.46 ± 0.06, *P* < 0.05; 0.39 ± 0.04, *P* < 0.05; and 0.32 ± 0.005, *P* < 0.0001, respectively) than in the group receiving only ETC-1002 (Figures 6(a) and 6(b)). The anti-inflammatory properties of ETC-1002 were also confirmed by the analysis of the mRNA expression of these three proinflammatory cytokines in the periodontium using RT-qPCR (Figure 6(c)). We found that the mRNA expression of IL-1*β*, IL-6, and TNF-*α* in the periodontitis group was, respectively, 2.59-, 5.23-, and 2.29-fold than that of the control group (Figure 6(c)). Additionally, the expression of these inflammatory cytokines was decreased in the ETC-1002 group, but was significantly increased in the ETC-1002+Compound C group (Figure 6(c)). These results again demonstrated that ETC-1002 exerts its anti-inflammatory activities through the AMPK signaling pathway.

**Figure 7:**
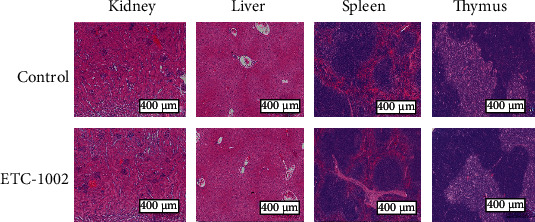
HE staining images of the kidney, liver, spleen, and thymus 10 days after the treatment with or without ETC-1002.

We also assessed ETC-1002 for possible toxicity *in vivo* by the histopathological observation of H&E-stained tissues of vital organs, including the kidneys, liver, spleen, and thymus. No abnormities were observed in any of the groups (Figure 7), indicating that ETC-1002 is safe for *in vivo* application.

## 4. Discussion

Periodontitis is a multifactorial chronic inflammatory disease of the periodontium [29]. In *Pg*-LPS-induced periodontitis, macrophages regulate the inflammatory response by producing a variety of proinflammatory mediators such as TNF-*α*, IL-6, and IL-1*β* [30–32]. The overproduction of these proinflammatory mediators induces connective tissue destruction and alveolar bone resorption, leads to distant organ damage, and causes or aggravates systemic diseases such as diabetes and cardiovascular disorders [33]. Accordingly, inhibiting the overproduction of these proinflammatory mediators is widely used as a means for screening anti-inflammatory agents. Small-molecule-based therapeutics continues to dominate the pharmaceutical landscape for the treatment of complex, multifactorial inflammatory conditions [34]. In this study, we found that ETC-1002 (molecular mass 344.49, <1000 Da), a small molecule drug, inhibited the *Pg*-LPS-induced production of the proinflammatory mediators: TNF-*α*, IL-6, and IL-1*β*, indicating that ETC-1002 may exhibit anti-inflammatory activity.

ETC-1002 is reported to be a potent activator of AMPK, a factor with a key role in the maintenance of cellular energy homeostasis and the negative regulation of inflammatory responses [35, 36]. Numerous *in vitro* and *in vivo* studies have shown that activating AMPK can result in a significant downregulation of the levels of proinflammatory cytokines, thereby exerting anti-inflammatory effects [34, 37]. In line with these results, we demonstrated that AMPK mediates the anti-inflammatory effects of ETC-1002. Specifically, ETC-1002 promoted the phosphorylation of AMPK*α in vitro* (Figure 3(a)) and inhibited the *Pg*-LPS-induced production of the proinflammatory mediators: TNF-*α*, IL-6, and IL-1*β*, in an AMPK-dependent manner (Figure 2). Meanwhile, in a murine model of periodontitis, ETC-1002 inhibited inflammatory responses, inflammatory cell infiltration, and the secretion of proinflammatory cytokines through AMPK signaling (Figures 5 and 6). These findings suggested that ETC-1002, a small molecule drug, through regulating the activation of AMPK, mimicked a cellular repair process without the adverse effects associated with traditional anti-inflammatory drugs and may represent a novel therapeutic agent for the treatment of inflammation-related diseases.

Based on the present findings, we propose a possible mechanism underlying the effects of ETC-1002 in *Pg*-LPS-stimulated RAW264.7 cells. The phosphorylation and consequent activation of AMPK have been shown to inhibit the NF-*κ*B signaling pathway [10]. NF-*κ*B is a transcription factor with a major role in the transcriptional regulation of genes involved in immune and inflammatory responses [38]. The most frequently occurring inducible form of NF-*κ*B consists of heterodimers of the p50/p65 subunits, which mainly function as transcriptional activators. Under normal conditions, NF-*κ*B exists in an inactive form in the cytoplasm, bound to I*κ*B*α*, an endogenous inhibitor [39]. When *Pg*-LPS, a microbial-associated molecular pattern (MAMP), binds to the pattern recognition receptors TLR-2/4 on the surface of macrophages [40], I*κ*B*α* is phosphorylated and subsequently degraded, allowing NF-*κ*B to translocate from the cytoplasm to the nucleus and the phosphorylation of p65, which contributes to the expression of proinflammatory mediators such as IL-1*β* and IL-6 [41, 42]. Accordingly, the inhibition of NF-*κ*B signaling is regarded as a potential target for the development of anti-inflammatory drugs. In this study, we demonstrated that ETC-1002 administration reduced the levels of phosphorylated I*κ*B*α* and p65, an effect that was significantly inhibited by pretreatment with AMPK*α*-targeting siRNA (Figure 3(b)). These findings suggested that ETC-1002 promoted the phosphorylation and activation of AMPK, thereby negatively regulating the activity of NF-*κ*B and, consequently, inhibiting inflammatory cytokine production and secretion and playing an anti-inflammatory role in the periodontitis.

These results are consistent with the idea that resolution of inflammation is an energy-requiring repair process [31]. As the metabolic control of inflammation constitutes the central principle of the associated repair mechanism, the design and development of new drugs that resolve inflammation by activating AMPK are urgent. TC-1002 may represent a novel therapeutic agent for the treatment of inflammation-related diseases and may help to reduce the adverse reactions associated with traditional anti-inflammatory drugs by simulating cellular processes essential for tissue repair in inflammation.

ETC-1002 is known to have multiple therapeutic targets, namely, the treatment of dyslipidemia, diabetes, and cardiometabolic disorders. Periodontitis is closely interrelated with systemic diseases. It has been suggested that “multitarget therapeutics is an attractive alternative strategy that could replace the prevailing ‘one drug one target' paradigm” [34]. This idea supports that ETC-1002 has potential as an anti-inflammatory drug suited to the treatment of periodontitis patients with systemic diseases.

The empirical results reported herein should be considered in the light of some limitations. First, the study focused on the anti-inflammatory effect of ETC-1002 in periodontitis, while the effect of ETC-1002 on bone resorption under the inflammatory microenvironment could be addressed in future research. Second, the experiment was still limited to animal experiment and clinical trials were highly needed.

## 5. Conclusions

In summary, we found that ETC-1002 exhibited excellent biocompatibility and notable potential as an anti-inflammatory drug *in vitro*. We further found that the AMPK/NF-*κ*B pathway contributed to the anti-inflammatory effects of ETC-1002 *in vitro*. Importantly, ETC-1002 could effectively inhibit periodontal tissue inflammation *in vivo* through AMPK signaling. Combined, our results suggested that ETC-1002 may have great potential for application as a treatment for periodontitis.

## Figures and Tables

**Table 1:** Primer sequences used for RT-qPCR.

Gene	Gene bank accession	Sequences of probes	Length (bp)	Product (bp)
IL-1*β*	NM_008361.4	F: TCCAGGATGAGGACATGAGCAC	22	105
		R: GAACGTCACACACCAGCAGGTTA	23	
IL-6	NM_001314054.1	F: GAGGATACCACTCCCAACAGACC	23	141
		R: AAGTGCATCATCGTTGTTCATACA	24	
TNF-*α*	NM_001278601.1	F: TATGGCCCAGACCCTCACA	19	151
		R: GGAGTAGACAAGGTACAACCCATC	24	
*β*-Actin	NM_007393.5	F: CATCCGTAAAGACCTCTATGCCAAC	25	171
		R: ATGGAGCCACCGATCCACA	19	

## Data Availability

The data used to support the findings of this study are available from the corresponding author upon reasonable request.
